# Stakeholder perceptions and public health system performance: evidence from Greece

**DOI:** 10.1093/eurpub/ckac131.552

**Published:** 2022-10-25

**Authors:** V Karokis-Mavrikos, M Mavrikou

**Affiliations:** 1 Department of Politics, University of Surrey, Surrey, UK; 2 Health Policy, Vianex, Athens, Greece

## Abstract

Policy choices, aims and priorities are defined by the dominant belief system among stakeholders in a policy area (Jenkins-Smith et al., 2018). Traditional assessments of health systems performance have focused on the institutional configurations, processes and resources in place (Murray and Frenk, 2000). We argue that the more latent dimension of ‘stakeholder perceptions’ is crucial to understanding why seemingly effective health system policy designs underperform during implementation. This paper brings in new evidence (2020) from an elite population study of 261 stakeholders in Public Health policy in Greece (politicians, civil servants, experts, interest groups, industry). We questioned stakeholders on: their conceptual understanding of public health, public health determinants, the field’s policy responsibilities and jurisdiction, threats and enabling factors to policy advancement, and public health systemic performance in Greece. Our findings highlight that: 1) stakeholder beliefs converge regarding drivers for effective policymaking - most prominently resources, expertise, coordination and management - but respondents also identify their scarcity in the Greek public health system (GPHS); 2) stakeholders converge regarding systemic pathogenies - most prominently managing inequalities and monitoring - and rank all legally stated functions of the GPHS poorly on average; 3) stakeholders agree that the existing paradigm in Greek public health policy does not promote the holistic perspective to health but most maintain a medicine-centric view of public health. We conclude that despite Greece enjoying a progressive public health system policy design, the mismatch between the system’s stated aims and the prevailing anachronistic stakeholder perceptions has hampered effective policy development. Future research should zero in on the importance of installing a co-oriented culture among stakeholders to achieve success in the performance of health systems.

**Key messages:**

• Stakeholder perceptions are crucial to understanding why seemingly effective health system policy designs under-perform during implementation.

• Despite Greece enjoying a progressive public health system policy design, the mismatch between the stated aims and the prevailing anachronistic stakeholder perceptions has hampered policy development.

